# Brain structural changes in women and men during midlife

**DOI:** 10.1016/j.neulet.2016.01.007

**Published:** 2016-02-26

**Authors:** J.Y. Guo, M. Isohanni, J. Miettunen, E. Jääskeläinen, V. Kiviniemi, J. Nikkinen, J. Remes, S. Huhtaniska, J. Veijola, P.B. Jones, G.K. Murray

**Affiliations:** a Department of Psychiatry, University of Cambridge, Box 189 Cambridge Biomedical Campus, CB2 0QQ, United Kingdom; b Department of Psychiatry, Institute of Clinical Medicine, P.O. Box 5000, 90014 University of Oulu, Oulu, Finland; c Department of Psychiatry, Oulu University Hospital, Kajaanintie 50, 90220 Oulu, Finland; d Institute of Health Sciences, P.O. Box 5000, 90014 University of Oulu, Oulu, Finland; e Unit of General Practice, Oulu University Hospital, Aapistie 1, 90220 Oulu, Finland; f Department of Diagnostic Radiology, Oulu University Hospital, Kajaanintie 50, 90220 Oulu, Finland; g Behavioural and Clinical Neuroscience Institute, University of Cambridge, Cambridge CB2 0QQ, United Kingdom

**Keywords:** NFBC 1966, Northern Finland Birth Cohort 1966, SIENA, structural image evaluation, using normalisation, FSL, FMRIB software library, FLIRT, FMRIB's linear image registration tool, FAST, FMRIB's automated segmentation tool, TFCE, threshold-free cluster enhancement, PBVC, percentage brain volume change, GM, grey matter, WM, white matter, CSF, cerebrospinal fluid, Longitudinal MRI, Gender difference, Sex differences, Sexual dimorphism, Ageing

## Abstract

Brain development during childhood and adolescence differs between boys and girls. Structural changes continue during adulthood and old age, particularly in terms of brain volume reductions that accelerate beyond age 35 years. We investigated whether brain structural change in mid-life differs between men and women. 43 men and 28 women from the Northern Finland 1966 Birth Cohort underwent MRI brain scans at age 33–35 (SD = 0.67) and then again at age 42–44 (SD = 0.41). We examined sex differences in total percentage brain volume change (PBVC) and regional brain change with FSL SIENA software. Women showed significant PBVC reduction compared with men between the ages of 33–35 and 42–44 years (Mean = −3.21% in men, Mean = −4.03% in women, *F* (1, 68) = 6.37, *p* < 0.05). In regional analyses, women exhibited greater brain reduction than men in widespread areas. After controlling for total percent brain volume change, men show greater relative regional brain reduction than women in bilateral precentral gyri, bilateral paracingulate gyri, and bilateral supplementary motor cortices. The results indicate sex differences in brain changes in mid-life. Women have more total brain reduction, and more reduction on the outer brain surface than men, whereas men exhibit more brain reduction on the mid-line surface than women after co-varying for total brain volume loss. These changes could contribute to sex differences in midlife behaviour and health.

## Highlights

• Women lost more total brain volume than men during 8.5 years follow-up in midlife.
• Women showed greater brain reduction in bilateral brain regions with and without co-varying for total brain volume loss.
• Men exhibited greater brain reduction in mid-line brain regions after correcting for the total brain volume loss.

## 1 Introduction

Brain structural differences between the sexes have been extensively investigated during the last several decades (reviewed by [1]). The most consistent finding so far is a larger brain size in men than in women (postmortem: [2]; in vivo imaging: [3]). Brain developmental trajectory differences between the sexes in children and adolescents have been cumulatively revealed by longitudinal studies ([4]; reviewed by Refs. [1], [5]). For example, a longitudinal paediatric neuroimaging study recruited 387 subjects between the ages of 3–27 years with 829 scans. The study reported that total brain volume peaked earlier in girls than in boys and that white matter increased during the age of 3–27 years in both boys and girls but with a faster rate of increase in boys in adolescence [6].

To date, little attention has been paid on whether brain structural changes differ between the sexes specifically during middle age. According to some previous cross-sessional post-mortem studies [7], [8], MRI studies [9], [10], [11] and a few longitudinal studies [12], brain structural changes tend to be more profound in men than in women during the lifespan. However, other studies have reported that there is no significant sex difference in brain structural changes, using a variety of study designs and examining various outcome measures such as global or regional measures (or both) [13], [14], [15], or indeed that regional brain structural changes are greater in women than in men during the lifespan. For instance, a multiple follow-up longitudinal study with 3 sets of scans 15 months apart reported a significant greater brain volume reduction in the pons in women than in men after the age of 49 years [16].

A recent review explored brain structural changes during the lifespan in healthy populations, including 56 longitudinal MRI studies and 2211 subjects, and revealed an inversed U shape of total brain volume loss across the lifespan. In particular, the study reported that total brain volume increased during childhood and adolescence, started to decrease since the ages of 13 years, and remained unchanged or even increased to certain extent during the ages of 18–35 years during young adulthood. After that, an accelerated total brain volume loss commenced from the age of 35 years with 0.2% total brain volume reduction annually. After the age of 60 years, total brain volume loss was more than 0.5% per year [17]. These results suggest that the brain volume changes are not linear in the healthy population during the lifespan; however, it remains unknown whether this acceleration in brain volume reduction affects men and women to the same extent.

To date, it is unknown if there are sex differences in brain volume changes during midlife, as the majority of studies are either cross-sectional or longitudinal focused on childhood or old age. Notably, there are significant disadvantages in drawing inferences of developmental processes, such as age related brain structural change, from cross-sectional studies [18], [19]. We had the opportunity to test whether brain structural change differs between the sexes during midlife in a representative birth cohort sample in which all participants were the same age and whom we followed longitudinally, providing an advantageous design to examine brain ageing.

## 2 Method

### 2.1 Participants

71 participants, all born in 1966, comprised the final sample for the present longitudinal study (43 men and 28 women, see Table 1). The participants were selected from the Northern Finland 1966 Birth Cohort (NFBC 1966), which is an unselected, general population based study [20]. The study was approved by the Ethical Committee of the Ostrobotnian Hospital District, Oulu, Finland; all participants provided written informed consent. Selection used truly random sampling from the birth cohort because we wanted to study a representative sample of the general population. The random sampling was gender stratified, because this group of general population volunteers was also intended to serve as a control for schizophrenia in a separate publication [21], [22], and schizophrenia has a higher incidence in men. 187 invitations were issued by letter, and 104 individuals took part in a baseline scan at age 33–35. The only exclusion criteria were contraindications to MRI, severe mental illness according to the national hospital discharge register (schizophrenia or other psychosis), inadequate scan quality of MRI diagnosis of pathology on neuroradiologist scan review. Of the original sample of 104 individuals, 100 individuals provided scans that passed quality control, and, of these, 77 returned an average of 9 years later for a follow-up MRI scan. One individual developed a psychotic episode and was excluded, and five additional participants were excluded due to inadequate scan quality.

### 2.2 The MRI scanner parameters

Brain MRI structural images from two time points in the present longitudinal study were collected by a GE Sigma 1.5 T MRI scanner in Oulu University Hospital. The baseline T1 weighted images were acquired using a three dimensional spoiled gradient echo (SPGR) sequence (slice thickness = 1.5 mm; in-plane resolution matrix size 256 × 256; voxel size 1.5 mm × 1 mm × 1 mm; TR = 35 ms; TE = 5 ms; and flip angle = 35°). Prior to the second time point the scanner was up-graded into HDxt with an 8 channel receiving coil. At follow-up T1 weighted images were acquired using the same parameters as before.

### 2.3 Calculation of percent brain volume change over time (PBVC)

We used the SIENA (Structural Image Evaluation, using Normalisation) [23] function in FSL (FMRIB Software Library) [24] to measure PBVC between baseline and follow-up time points for each participant. To this end, SIENA sequentially calls a series of FSL functions. BET (Brain Extraction Tool) extracts the brain and exterior skull from the non-brain tissue in the MRI head images for both time points. FLIRT (FMRIB's Linear Image Registration Tool) coregisters the two extracted brain images by using scaling and skewing transformations with constraint from the extracted exterior skull information. FAST (FMRIB's Automated Segmentation Tool) labels different brain tissue types and classifies them into two groups: grey matter (GM) and white matter (WM) into one group; background and cerebrospinal fluid (CSF) into another. GM and WM classified brain tissue volume are used to compute the percentage brain volume change. In particular, the outer and inner brain surfaces are defined by voxelwise brain edge points for both brain images from two time points. Then, PBVC over time for each participant is calculated by summarizing the perpendicular displacements along the image gradient direction between brain edges from two time points.

A number of registration based automated methods have been designed for estimating progressive brain atrophy using MR imaging in previous longitudinal studies, such as voxel-based morphometry (VBM) [25] and boundary shift integral (BSI) [26]. In the current longitudinal study, the scanner was up-traded into HDxt with a new gradient system with an 8 channel receiving coil prior to the follow-up time point, which led to our decision to use SIENA, as it is less sensitive to confounds arising from the difference pattern in image intensity histograms of the brain images from two time points, which is common after scanner upgrades [27]. In addition, SIENA has been widely used in longitudinal studies of neurological disorders and mental illnesses, such as Alzheimer's disease and dementia [28], [29], multiple sclerosis [30], [31] and normal aging [32]. In terms of reliability, previous studies have shown BSI and SIENA to have similar accuracy with around 0.2% error in estimating percentage brain volume change [33]; whereas SIENA exhibited a better accuracy than VBM with around 1.03% mean differences in estimating percentage brain volume change between two methods [34].

### 2.4 Regional brain change analysis

In order to explore regional brain change difference between the sexes, voxelwise SIENA (Structural Image Evaluation, using Normalisation) software in FSL (FMRIB Software Library) was applied in the present study. Voxelwise SIENA calculates regional brain change difference between the sexes by using brain edge displacements images for each participant generated in SINEA, namely flow images. The flow images for each participant are normalized into MNI152 standard space for multisubject statistics. Notably, as voxelwise SIENA measured brain edge displacements between two time points, the results of regional brain structural change difference in the sexes are purely restricted to the brain edge, and differs in this respect from classical voxel based morphometry.

### 2.5 Statistical analysis

PBVC values calculated by SIENA were exported into SPSS software for statistical analysis. We examined the effect of gender by using univariate analysis, adjusting for handedness. That is, the statistical model comprised one dependent variable (PBVC), one independent variable of interest (sex) and one covariate of no interest (handedness). By including (by chance) roughly 10% of left-handed participants in both sex groups, the present sample is reasonably representative of the general population. Handedness, therefore, was used as a covariate of no interest in the statistical analyses. It is noteworthy that the results remained the same when left-handed participants were excluded in the current study.

Regional brain change differences between men and women over time were explored by two statistical analyses using the permutation-based, voxelwise non-parametric testing tool Randomise in FSL [35], [36]. The first Randomise analysis examined which regions were contributing to this overall brain change by using one categorical independent variable (sex) and one categorical covariate (handedness). The second Randomise analysis investigated areas of regional brain change over and above overall change by applying one independent variable (sex) and two covariates (handedness and PBVC). Randomise produced statistical images using the Threshold-Free Cluster Enhancement (TFCE) function, correcting for multiple comparisons using family-wise error correction. For the purpose of reporting the results in Tables, the voxel-based maps of significant areas of change were divided into lobar regions based on anatomical atlases supplied with FSL (the MNI Structural Atlas for cortical regions and Harvard–Oxford Subcortical Structural Atlas for subcortical regions). Permutation tests are a type of non-parametric randomization test, which provides similar results to those obtained from a conventional general linear model in Statistical Parametric Mapping (SPM) [35]. This approach is preferable for experimental designs with low degrees of freedom, such as small sample sizes [37].

## 3 Results

### 3.1 Demographics and attrition analysis

Table 1 shows the demographic characteristics of the finalized 71 participants in the current study. In the final study sample (of study completers), there were no significant differences between men and women in age, interscan interval, handedness, education, or whether in fulltime employment at baseline (parental leave at the time of the base-line scan was coded as in employment as it was temporary in comparison to the period under study). The average age in men at the time of the baseline scan was 35.1 years, ranging from 33 to 35 years, and 43.6 years at follow-up, ranging from 42 to 44 years. The average age in women at baseline was 34.9 years (ranging from 32 to 35 years) and 43.4 at follow-up (ranging from 42 to 44 years). The average interscan interval was 8.5 years (ranging from 7.1 to 10.7) in men and in 8.61 years (ranging from 7.1 to 10.4) in women.

There was no significant difference in the ratio of men and women in the individuals who agreed to take part in the baseline MRI study (62 men, 42 women) compared to those who declined (54 men, 29 women; Chi-square = 0.58, *p* = 0.45). We examined whether those who completed the study (43 men, 28 women) differed from those who had a baseline scan but did not complete the study either due to drop-out or being excluded (19 men, 14 women). There were no significant differences between completers and non-completers in sex (Chi-square = 0.84, *p* = 0.77), or in level of education (basic, secondary, tertiary 3, 45, 23 in completers; 2, 18, 13 in non-completers; Chi-square = 0.77, *p* = 0.68), or whether in full-time work or not (61 yes, 10 not in completers; 24 yes, 9 not in non-completers; Chi-square = 2.62, *p* = 0.11).

### 3.2 Percent brain volume change difference between the sexes

**Fig. 1:**
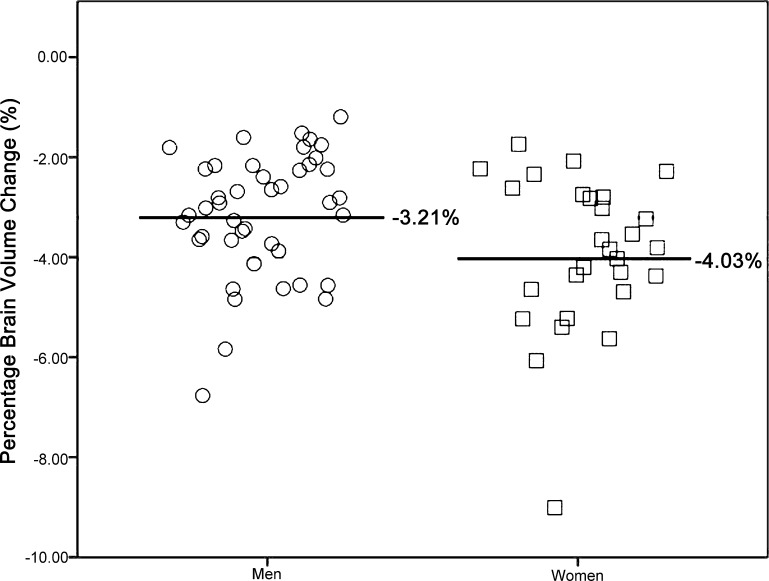
Percentage brain volume change by gender: women showed significantly greater total percentage brain volume loss than men between the ages of 34 to 43 years.

There was a significant (*F* (1, 68) = 6.37, *p* < 0.05) PBVC difference between men and women (controlling for handedness). There was greater PBVC decrease in women (Mean = −4.03%, SD = 1.54) than in men (Mean = −3.21%, SD = 1.17) between the ages of 34 to 43 years. As the scatter plot (Fig. 1) demonstrated that there was one outlier in the female group, we repeated the analysis with the outlier excluded even though there was no medical explanation for her outlier status: the results were unchanged.

### 3.3 Regional brain change difference between the sexes

**Fig. 2:**
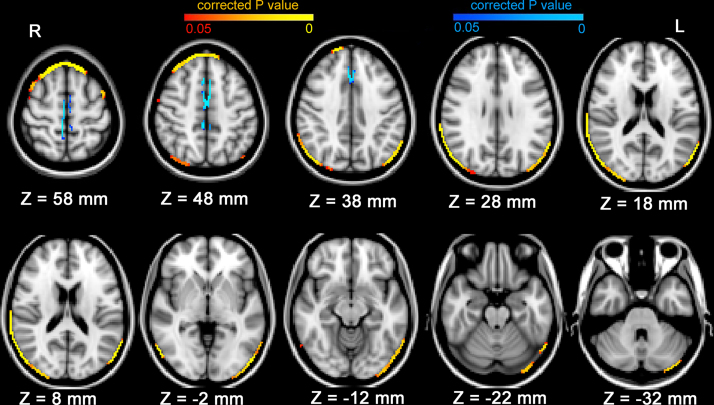
Sex differences in regional brain edge structural changes over time after controlling for total brain loss: women showed greater regional brain reduction compared with men on the edges of the bilateral frontal lobe, bilateral parietal lobe and bilateral occipital pole (shown in yellow and red), and less regional brain reduction than men on the edges of the bilateral precentral gyri, bilateral paracingulate gyri and supplementary motor cortices (shown in blue). (For interpretation of the references to color in this figure legend, the reader is referred to the web version of this article.)

The first Randomise analysis showed significantly (*p* < 0.05) greater regional brain reduction in women than in men (correcting for handedness) around the outer edge of the brain, including in the bilateral frontal, parietal, temporal, occipital cortex and cerebellum (Supplementary Table 1 and Figure). No differences were observed around the ventricular edge of the brain or midline edge of the hemispheres. The second Randomise analysis, which included the extra covariate PBVC, revealed significantly (*p* < 0.05) greater regional brain reduction in women than in men on the edge of bilateral frontal pole, bilateral superior frontal gyus, bilateral central gyrus, bilateral inferior parietal gyrus, left superior temporal gyrus (anterior division), right superior temporal gyrus (posterior division), bilateral middle temporal gyrus (tempero-occipital part), bilateral occipital lobe and left cerebellum (Supplementary Table 2 and Fig. 2). After correcting for the extra covariate PBVC, men exhibited significantly (*p* < 0.05) greater regional brain reduction than women in midline regions: on the edges of the bilateral precentral gyri, bilateral paracingulate gyri, and bilateral supplementary motor cortices (Supplementary Table 3 and Fig. 2).

## 4 Discussion

In the current longitudinal study, we found that women showed significantly greater brain volume reduction than men between the ages of 34 to 43 years. Women exhibited greater absolute regional brain reduction on the outer surfaces of the brain than men, When the total brain volume loss was corrected, women displayed greater brain reduction on the outer surfaces of the brain than men, whilst men showed significantly greater regional brain change reduction on the inner, midline surfaces of the brain than women. The putative mechanistic basis of these results, including the potential role of genetic and hormonal factors, is discussed in the Supplementary material.

### 4.1 Comparison with previous findings

The majority of longitudinal studies of brain ageing have focused on youth or old-age, with scant attention being paid to sex differences on brain ageing in mid-life. We are not aware of any other prospective, epidemiological, longitudinal studies to examine sex differences in brain structure change over a long period during mid-life. A recent large population based 4-years longitudinal study with 1172 participants of 65–82 years of age reported more gray matter atrophy in women than in men, where the annual rate of gray matter atrophy is −0.91% in women compared to −0.65% in men [38]. The observed difference between the sex-related rates of gray matter atrophy (−0.26%/year) in this study was slightly larger than PBVC difference found in the current study (−0.10%/year).

A variety of evidence indicates that brain development trajectories differ between the sexes in various stages of the lifespan. This work spans various research modalities, from pioneering results in experimental primate research [39], to the growing human neuroimaging literature. A recent review documented grey matter developmental trajectory differences in adolescence, with maximal cortical volumes peaking at a lower age in girls, and declining at a faster rate over adolescence in girls [5]. A cross-sectional study of 65 healthy individuals aged between 20 to 85 years revealed greater hippocampal and parietal lobe reduction in women than in men with increasing age [40]. Salinas et al. [41] explored cortical thickness in the parietal lobe in two age groups: children and adolescents with the ages of 7–17 years and an adult group with the ages of 18–47 years. They demonstrated that women showed significantly more parietal cortical thickness reduction in the adult group than in the young group, whereas men did not show a cortical thickness difference between the two age groups. Hatazawa et al. [42] also used a cross-sectional design to measure brain volume by the percentage of brain to cranial cavity ratio in 154 men and in 147 women with ages ranging from 20 to 79 years. They reported a non-linear trajectory of brain volume decreasing in both women and men. Specifically, the significant brain volume reduction began in the 60s in men and such decline continued steadily and gradually till the 80s, whereas such significant brain volume reduction commenced steadily in women at an earlier age, in the 50s, then starting another significant decrease from the 70s to the 80s. A notable finding from our study is the regional sexual dimorphism in progressive brain change, with prominent lateral atrophy in women and more medial atrophy in men.

Our findings are consistent with those of Cowell et al. in their study of sex differences in frontal lobe changes with age [43]: they found more prominent medial ageing effects in men and more prominent lateral ageing effects in women in their cross-sectional study. Our longitudinal study extends their results, showing that this dissociation between medial and lateral ageing effects is not confined to the frontal lobe. Thus, on review of the existing literature, there is ample evidence suggesting different trajectories of brain growth and atrophy between the sexes, and our study extends previous findings to show that women loss more brain volume than men, especially in lateral cortical areas, during mid-life.

### 4.2 Strengths and limitations

A strength of the study is its population base, which helps ensure the representativeness of the study. The longitudinal design is an advantage compared with the use of a cross-sectional study design when investigating ageing effects [44]. Specifically, Kraemer et al. [18] extensively discussed the disadvantages of using cross-sectional designs to draw longitudinal inferences, as this requires assumptions of a randomly selected sample, constant error variance, and parallel or fixed trajectories in participants. A limitation of the current study is the relatively short follow-up time (8.5 years) relative to the lifespan. However, in the context of existing longitudinal studies this follow-up period is very good. As in many longitudinal studies, scanner hardware upgrades provide a limitation, but an advantage of our study is the use of the FSL software tool SIENA, which is robust to changes in scanner hardware and software in estimating brain volume change over time and performs well in empirical evaluations in comparison to other available software tools [34]. A limitation is that 23 participants from our original sample of 100 failed to participate in the follow-up study, and that a further 6 volunteers were excluded due to image quality control and medical complications; it is difficult to completely eliminate attrition effects from long-term follow-up studies. Whilst the sample size is modest, this is mitigated to some extent by the fact that all the participants are from the same birth cohort, and thus are exposed to similar social and cultural influences, and importantly, are all the same age. Studies using participants of widely differing ages would need to be much larger in order to fully take into account age by gender interactions. A limitation of using the same age participants is that the results only apply to the mid-life period that we studied.

## 5 Conclusions

In a general population sample from Northern Finland, women lost more brain volume than men during midlife. Furthermore, midlife changes in women occur more extensively on the lateral edge of the brain, and in men more in midline structures. These findings may be relevant for understanding more about sex differences in behavior, personality and health in mid-life.

## Figures and Tables

**Table 1:** Demographic data.

	Men (*n* = 43)		Women (*n* = 28)		Statistics	Sig.
	Mean	S.D.	Mean	S.D.		
Age at baseline (years)	35.05	0.62	34.86	0.74	*F* = 1.29	.261
Age at follow-up (years)	43.55	0.41	43.47	0.42	*F* = 0.56	.459
Follow-up time (years)	8.50	0.63	8.61	0.72	*F* = 0.46	.501
Handedness (right/left)	40/3		26/2		*χ*^2^ = 0.00	.979
Education^a^	2/26/15		1/19/8		*χ*^2^ = 0.40	.818
Employment (yes/no)^b^	38/5		23/5		*χ*^2^ = 0.54	.461

a Education includes three levels ranging from elementary school (9 years or less), secondary school (10–12 years), or tertiary (over 12 years).

b Employment at the time of the base-line scan was coded as a dichotomised variable: full-time work or not. Maternity leave was considered as in employment.
