# Supplementary material for: Brain structural changes in women and men during midlife
Source: Neurosci Lett. 2016 Feb 26;615:107–12. doi: 10.1016/j.neulet.2016.01.007 (PMC4762229; doi:10.1016/j.neulet.2016.01.007)
Supplement: Supplementary file 1 [file mmc1.docx]

**Supplementary Material: Brain structural changes in women and men during midlife**

Guo JY^1^, Isohanni M^2,3^, Miettunen J^2,4,5^, Jääskeläinen E^2,3^, Kiviniemi V^6^, Nikkinen J^6^, Remes J^6^, Huhtaniska S^2^, Veijola J^2,3^, Jones PB^1,8^, Murray GK^1,8^

^1^Department of Psychiatry, University of Cambridge, Box 189 Cambridge Biomedical Campus, CB2 0QQ, United Kingdom

^2^Department of Psychiatry, Institute of Clinical Medicine, P.O. Box 5000, 90014 University of Oulu, Oulu, Finland.

^3^Department of Psychiatry, Oulu University Hospital, Kajaanintie 50, 90220 Oulu, Finland.

^4^Institute of Health Sciences, P.O. Box 5000, 90014 University of Oulu, Oulu, Finland

^5^ Unit of General Practice, Oulu University Hospital, Aapistie 1, 90220 Oulu, Finland.

^6^Department of Diagnostic Radiology, Oulu University Hospital, Kajaanintie 50, 90220 Oulu, Finland

^8^Behavioural and Clinical Neuroscience Institute, University of Cambridge, Cambridge CB2 0QQ, UK.

Correspondence to: Dr Murray, gm285@cam.ac.uk, Department of Psychiatry, University of Cambridge, Box 189 Cambridge Biomedical Campus, CB2 0QQ, United Kingdom. +441223769499

**Supplementary Results**

**Supplementary Table 1 Regional brain change results in women and men without correcting for total percentage brain volume change over 8.5 years**

| Clusters | Group | Mean of edge Displacement (mm) | | Number of Voxels | t | P (peak) | Coordinate of peak voxel (MNI152mm) | | |
| --- | --- | --- | --- | --- | --- | --- | --- | --- | --- |
|  |  | Mean | SD |  |  |  | x | y | z |
| Frontal Lobe | Women | -0.27 | 0.12 | 5085 | 3.17 | .005 | -12 | 42 | 52 |
|  | Men | -0.09 | 0.11 |  |  |  |  |  |  |
| Parietal Lobe | Women | -0.25 | 0.13 | 3608 | 3.29 | .006 | 58 | -68 | 12 |
|  | Men | -0.08 | 0.09 |  |  |  |  |  |  |
| Temporal Lobe | Women | -0.29 | 0.09 | 1329 | 3.18 | .005 | 60 | -66 | 10 |
|  | Men | -0.16 | 0.09 |  |  |  |  |  |  |
| Occipital Lobe | Women | -0.39 | 0.15 | 2902 | 3.25 | .005 | 58 | -72 | 0 |
|  | Men | -0.20 | 0.16 |  |  |  |  |  |  |
| Cerebellum | Women | -0.37 | 0.18 | 1008 | 2.69 | .012 | -34 | -88 | -32 |
|  | Men | -0.20 | 0.17 |  |  |  |  |  |  |

^a^ atlas: Widespread cortical brain changes were parcellated into regions using the MNI Structural Atlas; statistical parameters were extracted from Randomise results.

Supplementary Table 2 Regional brain change results in women and men after correction for total percentage brain volume change over 8.5 years (women greater loss than men)

| Clusters | Group | Mean of edge Displacement (mm) | | Number of Voxels | t | p (peak) | Coordinate of peak voxel (MNI152mm) | | |
| --- | --- | --- | --- | --- | --- | --- | --- | --- | --- |
|  |  | Mean | SD |  |  |  | x | y | z |
| Bilateral Frontal Pole | Women | -0.25 | 0.16 | 799 | 3.36 | .008 | -12 | 40 | 54 |
|  | Men | -0.02 | 0.18 |  |  |  |  |  |  |
| Bilateral SFG | Women | -0.36 | 0.15 | 877 | 3.25 | .007 | -14 | 30 | 60 |
|  | Men | -0.15 | 0.15 |  |  |  |  |  |  |
| Bilateral central gyrus | Women | -0.33 | 0.19 | 1046 | 2.99 | .013 | 30 | -4 | 70 |
|  | Men | -0.13 | 0.14 |  |  |  |  |  |  |
| Bilateral IPG | Women | -0.18 | 0.12 | 215 | 3.42 | .008 | 68 | -42 | 26 |
|  | Men | 0 | 0.14 |  |  |  |  |  |  |
| Left STG (AD) | Women | -0.44 | 0.19 | 16 | 4.01 | .042 | -64 | -2 | 2 |
|  | Men | -0.23 | 0.17 |  |  |  |  |  |  |
| Right STG (PD) | Women | -0.16 | 0.13 | 126 | 3.24 | .012 | 68 | -40 | 26 |
|  | Men | 0.02 | 0.15 |  |  |  |  |  |  |
| Bilateral MTG (TOP) | Women | -0.33 | 0.11 | 494 | 3.40 | .011 | 58 | -68 | 12 |
|  | Men | -0.15 | 0.10 |  |  |  |  |  |  |
| Bilateral Occipital Lobe | Women | -0.37 | 0.15 | 2264 | 3.33 | .011 | 58 | -72 | 12 |
|  | Men | -0.14 | 0.13 |  |  |  |  |  |  |
| Left Cerebellum | Women | -0.35 | 0.22 | 188 | 2.95 | .016 | -34 | -88 | -28 |
|  | Men | -0.13 | 0.19 |  |  |  |  |  |  |

^a^ abbreviations: SFG = superior frontal gyrus; IPG = inferior parietal gyrus; STG (AD) = superior temporal gyrus (anterior division); STG (PD) = superior temporal gyrus (posterior division); MTG (TOP) = middle temporal gyrus (temporooccipital part).

^b^ atlas: Regional brain changes were parcellated according to Harvard-Oxford Cortical Structural Atlas; statistical parameters were extracted from Randomise results.

Supplementary Table 3 Regional brain change results in women and men after correction for total percentage brain volume change over 8.5 years (men greater loss than women)

| Clusters | Group | Mean of edge Displacement (mm) | | Number of Voxels | t | p (peak) | Coordinate of peak voxel (MNI152mm) | | |
| --- | --- | --- | --- | --- | --- | --- | --- | --- | --- |
|  |  | Mean | SD |  |  |  | x | y | z |
| Bilateral precentral gyri | Women | -0.08 | 0.05 | 400 | 3.78 | .015 | 6 | -36 | 56 |
|  | Men | -0.13 | 0.06 |  |  |  |  |  |  |
| Bilateral PG | Women | -0.10 | 0.07 | 264 | 3.76 | .013 | -4 | 10 | 50 |
|  | Men | -0.14 | 0.08 |  |  |  |  |  |  |
| Bilateral SMC | Women | -0.08 | 0.05 | 243 | 3.77 | .012 | -2 | -2 | 50 |
|  | Men | -0.14 | 0.08 |  |  |  |  |  |  |

^a^ abbreviations: PG = paracingulate gyrus; SMC = supplementary motor cortices.

^b^ atlas: Regional brain changes were parcellated using the Harvard-Oxford Cortical Structural Atlas for cortical regions; statistical parameters were extracted from Randomise results.


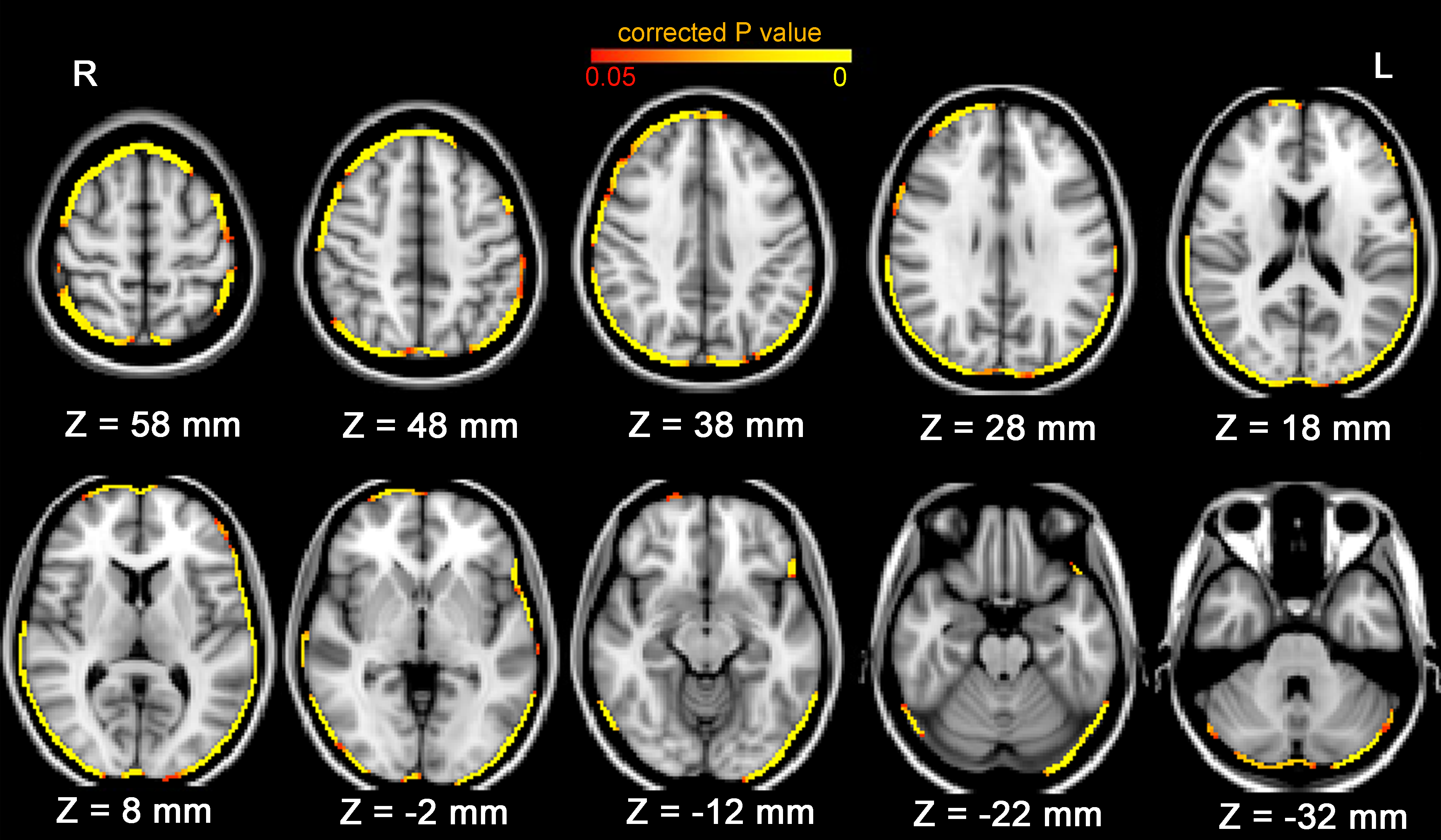


Supplementary Figure. Sex differences in regional brain edge structural changes over time: women displayed significantly (p < .05, family-wise error corrected) greater reduction across a wide expanse of the outer brain edge than men (adjusted for handedness but not for global brain volume change).

**Supplementary Discussion: Theoretical explanations**

Hormonal differences are strong candidates to explain underlying sex differences in brain structural change over time ([Peper et al., 2011](#_ENREF_36), [Bramen et al., 2012](#_ENREF_2), [Nguyen et al., 2013](#_ENREF_33)). There is evidence for testosterone having a neuroprotective effect in some circumstances (Tehranipour & Moghimi 2010), which could potentially contribute to our findings. Neuroimaging evidence has pointed to a possible causal relationship between testosterone levels and brain morphology; a longitudinal study examined testosterone-related cortical thickness changes in children with the ages of 4 to 22 years and reported that females exhibited opposite correlations between testosterone levels and cortical thickness before and after puberty, whereas males showed persistent negative correlations of testosterone levels and cortical thickness throughout puberty ([Nguyen et al., 2013](#_ENREF_33)).

Alternatively, or additionally, it is possible to speculate that hormonal changes related to midlife reproductive decline could contribute to the differential loss of brain volume we observed in women. Notably, the reproductive decline becomes sharper in women than in men during the midlife, which may lead to different influences of reproductive hormones on brain structural changes in men and women ([MacNaughton et al., 1992](#_ENREF_29), [Ottinger, 2010](#_ENREF_35)). Whilst the average age for the menopause in Finland at the time of our study was 50-51 (Pakarinen et al., 2010), previous research suggests that by age 45, about 4% of women will be postmenopausal and about 20% of women will be “menopausal” (operationally defined as having had a period within the last year but not within the last three months) (McInley et al 1972). Research from animal models suggests estrogen modulates synaptic plasticity and has neuroprotective properties (Bryant et al., 2006; Morrison et al., 2006), which is consistent with emerging experimental evidence in humans, albeit in small sample sizes (e.g. [Raz et al., 2004](#_ENREF_40)).

Estrogens have been shown to be pro-cognitive, lending further evidence to the thesis that reproductive decline in women may link with neural changes ([Koebele and Bimonte-Nelson, 2015](#_ENREF_28)). For instance, estradiol treatment promotes improvement of cognitive function, such as selective attention and working memory ([Baker et al., 2012](#_ENREF_2)) and short term memory ([Krug et al., 2006](#_ENREF_30)). Furthermore, estrogen may be protective against deleterious effects of glucocorticoids on the brain, potentially mitigating against stress induced harms ([Ycaza Herrera and Mather, 2015](#_ENREF_56)).

For the interpretation of our study results, it may be significant that the hormonal changes that lead to the menopause begin many years prior to the final menstrual period, with some relevant changes occurring as an ongoing process throughout the reproductive lifespan. For example, whilst the changes in estrogen levels are relatively restricted to the time relatively close to the menopause, there is a much longer gradual decline in inhibin and rise in follicle stimulating hormone that is thought to proceed from menarche onwards (Santoro 2005).

Genetic factors are likely to also play an important role in sex differences in brain structural trajectories. It is well established that brain morphology is highly heritable, and whilst the precise molecular genetic basis of measures such as total brain volume or regional brain volumes remains yet to be elucidated, some recent progress has been made (Hibar et al., 2015, Strike et al., 2015). For example, Raznahan and colleagues showed that variation in length of a functional polymorphic trinucleotide repeat within the gene for the androgen receptor is associated with differential changes in cortical thickness during adolescence (Raznahan et al., 2010). Emerging evidence indicates that gene expression changes in the brain across the lifespan are sexually dimorphic (Berchtold et al., 2008). For instance, one study examined 44 individuals aged between 20 to 99 years, and found 667 genes expressed in superior frontal gyrus that showed significant heterochrony between the sexes (about 5% of the transcriptome), of which 654 (98%) heterochronic genes revealed earlier and/or faster changes in women than in men ([Yuan et al., 2012](#_ENREF_52)). Such sex differences in heterochronic gene expression has been proposed as a putative mechanism to explain why women show higher age-specific risks of dementia than men (Yuan et al., 2012).

Baker LD, Asthana S, Cholerton BA, Wilkinson CW, Plymate SR, Green PS, Merriam GR, Fishel MA, Watson GS, Cherrier MM, Kletke ML, Mehta PD, Craft S (2012) Cognitive response to estradiol in postmenopausal women is modified by high cortisol. Neurobiol Aging 33:829 e829-820.

Bramen JE, Hranilovich JA, Dahl RE, Chen J, Rosso C, Forbes EE, Dinov ID, Worthman CM, Sowell ER (2012) Sex matters during adolescence: testosterone-related cortical thickness maturation differs between boys and girls. PLoS One 7:e33850.

Berchtold NC, Cribbs DH, Coleman PD, Rogers J, Head E, Kim R, Beach T, Miller C, Troncoso J, Trojanowski JQ, Zielke HR, Cotman CW (2008) Gene expression changes in the course of normal brain aging are sexually dimorphic. Proc Natl Acad Sci U S A. Oct 7;105(40):15605-10.

Bryant DN, Sheldahl LC, Marriott LK, Shapiro RA, Dorsa DM (2006) Multiple pathways transmit neuroprotective effects of gonadal steroids. *Endocrine* 29:199–207.

Hibar DP, Stein JL, Jahanshad N, Kohannim O, Hua X, Toga AW, McMahon KL, de Zubicaray GI, Martin NG, Wright MJ; Alzheimer's Disease Neuroimaging Initiative, Weiner MW, Thompson PM (2015) Genome-wide interaction analysis reveals replicated epistatic effects on brain structure. Neurobiol Aging 36 Suppl 1:S151-8.

Koebele SV, Bimonte-Nelson HA (2015) Trajectories and phenotypes with estrogen exposures across the lifespan: What does Goldilocks have to do with it? Horm Behav 74:86-104.

Krug R, Born J, Rasch B (2006) A 3-day estrogen treatment improves prefrontal cortex-dependent cognitive function in postmenopausal women. Psychoneuroendocrinology. 31:965-75.

McKinley S, Jefferys B, Thompson B (1972) An investigation of the age at menopause J. Biosoc. Sci., 4: 161–173.

MacNaughton J, Banah M, McCloud P, Hee J, Burger H (1992) Age related changes in follicle stimulating hormone, luteinizing hormone, oestradiol and immunoreactive inhibin in women of reproductive age. Clin Endocrinol (Oxf) 36:339-345.

Morrison JH, Brinton RD, Schmidt PJ, Gore AC (2006) Estrogen, menopause and the aging brain: how basic neuroscience can inform hormone therapy in women J. Neurosci., 26: 10332–10348

Ottinger MA (2010) Mechanisms of reproductive aging: conserved mechanisms and environmental factors. Ann N Y Acad Sci 1204:73-81.

Nguyen TV, McCracken J, Ducharme S, Botteron KN, Mahabir M, Johnson W, Israel M, Evans AC, Karama S (2013) Testosterone-related cortical maturation across childhood and adolescence. Cereb Cortex 23:1424-1432.

Pakarinen M, Raitanen J, Kaaja R, Luoto R (2010) Secular trend in the menopausal age in Finland 1997–2007 and correlation with socioeconomic, reproductive and lifestyle factors. Maturitas 66:417-422.

Peper JS, van den Heuvel MP, Mandl RC, Hulshoff Pol HE, van Honk J (2011) Sex steroids and connectivity in the human brain: a review of neuroimaging studies. Psychoneuroendocrinology 36:1101-1113.

Raz N, Rodrigue KM, Kennedy KM, Acker JD (2004) Hormone replacement therapy and age-related brain shrinkage: regional effects. Neuroreport 15:2531-2534.

Raznahan A, Lee Y, Stidd R, Long R, Greenstein D, Clasen L, Addington A, Gogtay N, Rapoport JL, Giedd JN (2010). Longitudinally mapping the influence of sex and androgen signaling on the dynamics of human cortical maturation in adolescence. Proc Natl Acad Sci U S A. 107:16988-93.

Santoro N (2005) The menopausal transition. Am J Med. 2005 118 Suppl 12B:8-13.

[Strike LT](http://www.ncbi.nlm.nih.gov/pubmed/?term=Strike%20LT%5BAuthor%5D&cauthor=true&cauthor_uid=25773500), [Couvy-Duchesne B](http://www.ncbi.nlm.nih.gov/pubmed/?term=Couvy-Duchesne%20B%5BAuthor%5D&cauthor=true&cauthor_uid=25773500), [Hansell NK](http://www.ncbi.nlm.nih.gov/pubmed/?term=Hansell%20NK%5BAuthor%5D&cauthor=true&cauthor_uid=25773500), [Cuellar-Partida G](http://www.ncbi.nlm.nih.gov/pubmed/?term=Cuellar-Partida%20G%5BAuthor%5D&cauthor=true&cauthor_uid=25773500), [Medland SE](http://www.ncbi.nlm.nih.gov/pubmed/?term=Medland%20SE%5BAuthor%5D&cauthor=true&cauthor_uid=25773500), [Wright MJ](http://www.ncbi.nlm.nih.gov/pubmed/?term=Wright%20MJ%5BAuthor%5D&cauthor=true&cauthor_uid=25773500) (2015) Genetics and brain morphology. Neuropsychol Rev. 25:63-96.

Tehranipour M, Moghimi A (2010) Neuroprotective effects of testosterone on regenerating spinal cord motoneurons in rats. J Mot Behav 42:151-5.

Ycaza Herrera A, Mather M (2015) Actions and interactions of estradiol and glucocorticoids in cognition and the brain: Implications for aging women. Neurosci Biobehav Rev 55:36-52.

Yuan Y, Chen YP, Boyd-Kirkup J, Khaitovich P, Somel M (2012) Accelerated aging-related transcriptome changes in the female prefrontal cortex. Aging Cell 11:894-901.
